# Solar-induced hybrid energy harvesters for advanced oxidation water treatment

**DOI:** 10.1016/j.isci.2021.102808

**Published:** 2021-07-01

**Authors:** Zheng-Yang Huo, Dong-Min Lee, Young-Jun Kim, Sang-Woo Kim

**Affiliations:** 1 School of Advanced Materials Science and Engineering, Sungkyunkwan University (SKKU), Suwon 16419, Republic of Korea; 2 SKKU Advanced Institute of Nanotechnology (SAINT), Samsung Advanced Institute for Health Sciences & Technology (SAIHST), Sungkyunkwan University (SKKU), Suwon 16419, Republic of Korea

**Keywords:** Energy Resources, Energy sustainability, Energy systems

## Abstract

Water treatment based on advanced oxidation processes (AOPs) supplies clean water to rural areas lacking electric power supply and/or during natural disasters and pandemics. Considering the abundance of solar energy in the ambient environment, the solar-driven AOPs show an interesting potential to driving the water purification process. Involving the energy harvester (EH) that harvests mechanical or thermal energy into electricity to the solar-driven AOPs can achieve sustainable and self-powered water purification. Herein, we summarize the recent progress in the application of solar-induced hybrid EHs that harvest solar and mechanical/thermal energy simultaneously to drive AOP water treatment. A detailed discussion of the solar-induced hybrid EHs enabling AOP water treatment based on the mechanisms of piezo-, tribo-, pyro-, and thermo-assisted photocatalysis is provided. In addition, this paper explores future opportunities and strategies of the solar-induced hybrid EHs to drive the AOP water treatment in actual situations with unstable and fluctuating environmental conditions.

## Graphical abstract

**Figure:**
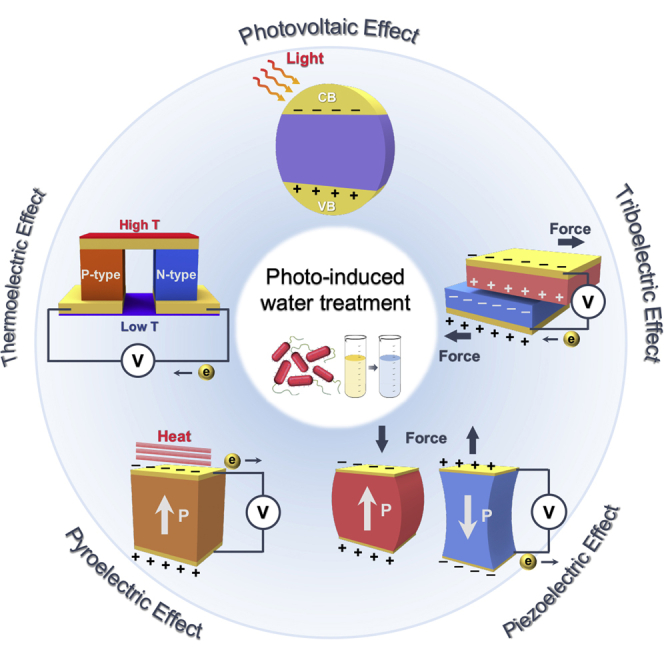

## Introduction

Currently, nearly a third of the world population does not have reliable access to clean water due to its finite nature and pollution vulnerability (Alvarez et al., 2018; Chu et al., 2019; Westerhoff et al., 2019). The situation has been aggravated by the rapid industrialization, population growth, and urbanization; these three pose a significant water pollution threat through the discharge of organic pollutants including pharmaceuticals, endocrine disruptors, chemical additive/solvents, and human and domestic waste (Bach et al., 2012; Cantwell et al., 2018; Rice and Westerho, 2017). These pollutants could be non-biodegradable, toxic, and recalcitrant under ambient conditions, and, therefore, a huge potential threat to public health (Hodges et al., 2018; Miklos et al., 2018). In addition, water pollution provides pathogenic microbes with favorable thriving conditions, therefore, increasing the risk of waterborne diseases, morbidity, and mortality (Huo et al., 2020; Song et al., 2016; Wang et al., 2019b). Other factors that contribute to the scarcity of clean water are lack of adequate sanitation and shortage of electric power supply, which is required for water purification; this is especially in the rural areas and in developed areas that are facing pandemics/disasters (e.g., COVID-19) (Ahmed et al., 2020; Bogler et al., 2020; Huo et al., 2020).

Water treatment is a feasible solution for water pollution and drinking water shortage. The widening gap between water pollution and treatment has obliged a paradigm shift from the conventional centralized to the emerging decentralized water treatment. Unlike a centralized water treatment system with large pipe network transportation, a decentralized treatment system enables water purification within a smaller scale and closer to its point of consumption (Chaplin, 2019; Huo et al., 2016; Liu et al., 2019b; Pooi and Ng, 2018). As a result, a decentralized system is cost-effective, fast, and energy-efficient, therefore a suitable option for developing countries, as well as regions facing natural disasters or pandemics.

Advanced oxidation processes (AOPs) are appealing treatment options for such decentralized systems (Hodges et al., 2018; Miklos et al., 2018; Xing et al., 2018). AOPs are designed to remove organic pollutants and microbes in water by oxidation. Pollutants can be oxidized to low-toxic or non-toxic molecules under specific reaction conditions *in-situ* generated reactive oxygen species (ROS) with high oxidation ability including ·OH, H_2_O_2_, ·O_2_^-^, and ·Cl (Boczkaj and Fernandes, 2017; Li et al., 2018; Moreira et al., 2017). Assisted by the sharply developed novel material fabrication (i.e., catalysis design) and device construction methods, AOPs can achieve high-efficiency ROS generation enabling fast organic pollutant degradation and microbial disinfection (Wang et al., 2019a, 2019b, 2019c). There are several AOPs mechanisms and the most common in water purification are peroxy-based, light-driven, and electric-driven AOPs. In the peroxy-based water treatment mechanism, some specific weak oxidants (i.e., Fe^2+^/Fe^3+^ and persulfates) are triggered by H_2_O_2_ or metal oxides catalyst under certain conditions to generate ROS for water purification (Qi et al., 2018; Zhang et al., 2017a). However, due to its substantial chemical demand, its application for decentralized water treatment is limited. In the light-driven AOP treatment, ROSs are generated through photocatalysis (Liu et al., 2016, 2017; Loeb et al., 2019). An electric-driven AOP mechanism uses electric energy (either from on-grid electricity or solar cell) to produce ROS for pollutant degradation and microbial disinfection through an electrochemical process (Lin et al., 2018; Liu et al., 2019a; Yang, 2020). With adequate solar energy in rural areas, light-driven and solar cells-powered electric-driven AOPs are the most suitable options for decentralized water treatment (Cheng et al., 2020; Chu et al., 2019; Nie et al., 2016). However, solar energy is unreliable due to sunlight fluctuation and overdependence, which led to a large construction footprint, and additional costs from installing storage devices (e.g., battery and capacitor) during solar energy utilization (Guerin, 2017; Zhang et al., 2018).

**Figure 1:**
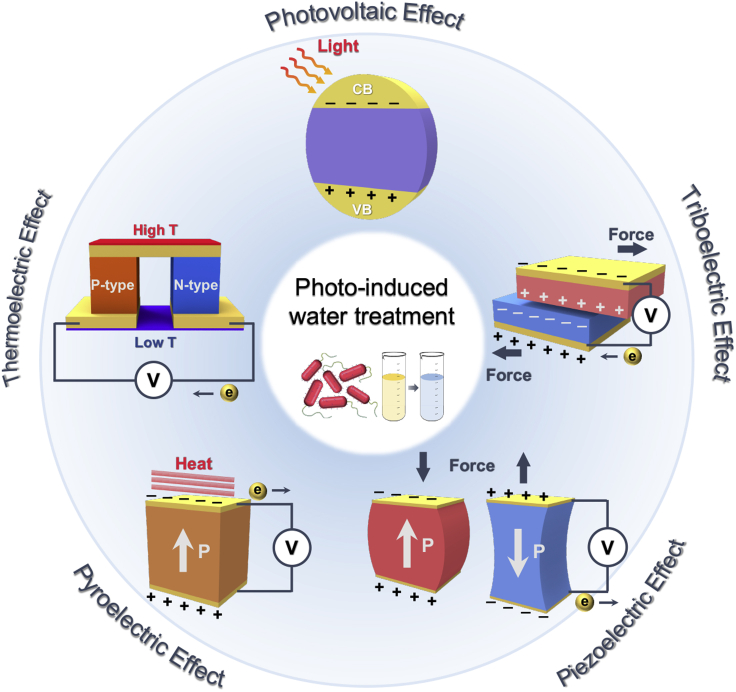
Schematics of solar-induced hybrid energy harvesters (EHs) harnessing mechanical energy based on piezoelectric and triboelectric effect and thermal energy based on pyroelectric and thermoelectric effect For advanced oxidation processes (AOPs) based water purification, solar energy mainly leads to photo-excitation. The charge carrier electrons exist in the conduction band (CB), the charge carrier holes exist in the valence band (VB), and the two bands (CB and VB) are separated to generate polarization when with solar irradiation. Insert images indicate potential applications for water treatment based on AOPs including microbial disinfection and pollutant degradation.

Besides solar energy, other green energies such as wind and flow kinetic, wave, heat, and vibrations energies are promising options. With proper management using energy harvesting systems, these can be converted into electric energy and provide continuous and sustainable green power for decentralized water treatment (Cao et al., 2016; Chen et al., 2016; Park et al., 2016). Energy harvesters (EHs) are based on different energy conversion mechanisms, and the most common harvesting mechanisms are piezoelectric, triboelectric, thermoelectric, and pyroelectric effects (Park et al., 2014; Pu et al., 2015; Rojas et al., 2017; Xia et al, 2018, 2020b; Zi et al., 2015). Although researchers have confirmed the feasibility of EHs to drive the AOPs based water treatment systems, they commonly rely on a single energy source, and therefore are still limited by unstable and fluctuating ambient conditions in practical applications (e.g., weather, temperature, and humidity) (Ding et al., 2019; Thuy Phuong et al., 2020; Tian et al., 2017; Xia et al., 2020c). This necessitated the development of a solar-induced hybrid EHs, a hybrid system that integrates mechanical and thermal EHs into solar EHs enabling simultaneous energy harvesting and continuous energy conversion (Figure 1). The hybrid EHs are sustainable and therefore a promising energy system for the AOPs water treatment process (Abdelraheem et al., 2020; De la Obra Jiménez et al., 2019; Rizzo et al., 2019).

In the solar-induced hybrid EH-powered AOPs, sunlight can be the major energy source during the daytime while at night and rainy/cloudy days, mechanical or thermal energy harvesting systems can be the major sources of energy for the AOP water treatment system. In addition, when the mechanical or thermal energy harvesting systems are integrated into the solar-driven AOP water treatment system, new synergy mechanisms may be developed resulting in a higher ROS generation efficiency and therefore better treatment performance (Qian et al., 2020; Zhang et al., 2017b).

Here, we summarized the recent research progress of the solar-induced hybrid EHs and their application in AOP water treatment systems. We have provided a summary of different types of solar-induced hybrid EHs for AOP water treatment based on the energy harvesting mechanisms including: (1) solar-induced piezoelectric effect, (2) solar-induced triboelectric effect, (3) solar-induced pyroelectric effect, and (4) solar-induced thermoelectric effect. Detailed studies on solar-induced hybrid EHs for pollutant degradation and microbial disinfection with enhanced, reliable, and robust performances are described. This review also provides future opportunities and perspectives for the practical application of solar-induced hybrid EHs during unstable and fluctuating environmental conditions.

## Solar-induced hybrid EHs for AOP water treatment

Solar-induced hybrid EHs for continuous energy harvesting has continued to grow as promising candidates to drive the AOP water treatment processes owing to their high performance and coupling effect between solar and other energy harvesting systems including mechanical and thermal EHs (Chou et al., 2019; Pan et al., 2020; Yang et al., 2013a). Mechanical energy harvesting systems consist of piezoelectric and triboelectric-based EHs, while thermal energy harvesting systems consist of pyroelectric and thermoelectric-based EHs (Lee et al., 2016; Ryu et al., 2019). The energy harvesting mechanism and the application of AOP water treatment using solar-induced hybrid EHs are summarized below.

### Solar-induced piezoelectric EHs for AOP water treatment

**Figure 2:**
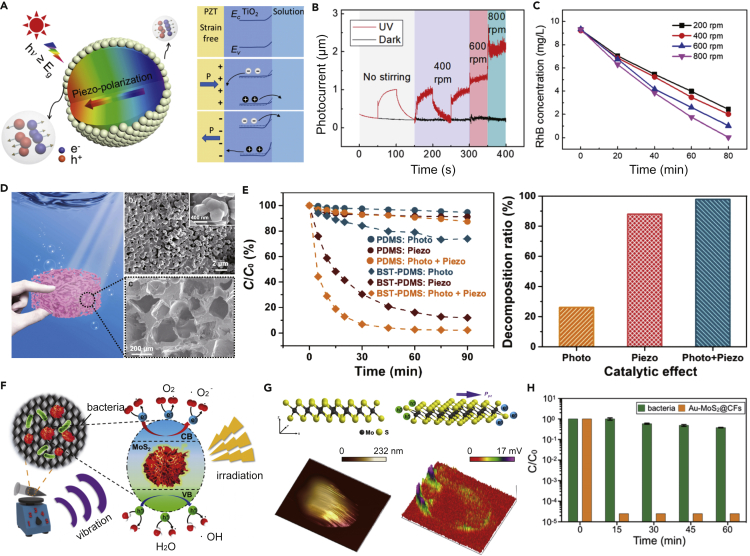
Emerging applications using solar-induced piezoelectric EHs for AOP water treatment (A) Schematics showing the formation of the piezoelectric field in the lead zirconate titanate (PZT), and illustrations of band structure diagrams of TiO_2_ at the photocatalyst-solution interface with the piezoelectric-potential. (B) Photocurrent output on PZT/TiO_2_ electrode with solar irradiation and water stirring. (C) Organic pollutant (Rhodamine B; RhB) degradation efficiency using PZT/TiO_2_ with solar irradiation and water stirring, showing an enhanced performance degradation efficiency based on the piezo-assisted photocatalysis mechanisms. Reproduced with permission from (Feng et al., 2018), Copyright 2018, American Chemical Society. (D) Schematics showing the barium strontium titanate (BST)-PDMS porous foam for piezo-assisted photocatalysis degradation. (E) RhB degradation efficiency using BST-PDMS porous foam with periodic mechanical squeezing and solar irradiation. Reproduced with permission from (Xu et al., 2020), Copyright 2020, Elsevier Ltd. (F) Schematics of the MoS_2_ nanocatalyst for AOP water disinfection. The MoS_2_ nanocatalyst can be triggered to generate reactive oxygen species (ROS including ·OH and ·O_2_^-^) by mechanical vibration (piezoelectric effect) and light irradiation (photocatalytic effect). (G) Schematics showing MoS_2_ with an external force, and illustrations of piezoelectric polarization at active edge sites under an external force. (H) Disinfection performance of Au-MoS_2_@CFs under a combination of mechanical vibration and solar irradiation. Reproduced with permission from (Chou et al., 2019), Copyright 2019, Elsevier Ltd.

The piezoelectric EH converts mechanical energy in the ambient environment including wind/water flow, waves, and mechanical vibration into electric energy (Han et al., 2018; Kim et al., 2020a; Yoon and Kim, 2020). With specific features that generate electric charges in response to applied mechanical stress, the piezoelectric materials can form internal potential on the surfaces of the material (Lee et al., 2013, 2015; Thuy Phuong et al., 2020). Utilizing this feature, where piezoelectric effect into photocatalysts is involved under the simultaneous photo irradiation and external mechanical stress, new solar-induced piezoelectric EHs will be developed and applied for AOP water treatment (Chen et al., 2017; Pan et al., 2020). Feng et al. developed a Titania (TiO_2_) modified lead zirconate titanate (PZT) catalyst with a core-shell structure by coating TiO_2_ nanoparticles on the surface of piezoelectric PZT microsphere (Feng et al., 2018). During the water treatment process, the stirring process exerts external stress on the PZT/TiO_2_, which leads to a piezoelectric field in the PZT core structure. Meanwhile, when UV light irradiation falls on the outer TiO_2_ semiconductor, photoelectrons and holes are generated due to the photo-excitation, and the generated photoelectrons and holes are separated without recombination by promotion from the piezo-induced bias voltage (Figure 2A). This specific mechanism leads to enhanced quantum efficiency and charge generation performance. Prompted by the piezo-assisted photocatalysis activity, the generated holes react with water to form hydroxyl radicals (·OH), and the photoelectrons react with oxygen to form superoxide anions (·O_2_^-^). The generated ROSs finally enables organic pollutant degradation in the water (Figure 2A).

The transient photocurrent measurement was carried out to investigate the photocatalytic performance of the PZT/TiO_2_ particles. As shown in Figure 2B, without light (in dark conditions), there was no notable current output from the PZT/TiO_2_, even with the water-stirring operation. When UV irradiation was applied, significant photocurrent output was generated (0.79 μA) without the water-stirring, which indicated the UV-response of the PZT/TiO_2_ electrode. When both water stirring and UV irradiation were applied, the amount of photocurrent generated by PZT/TiO_2_ particles was significant and continued to increase with the water-stirring rate. At the water-stirring rate of 600 rpm, the photocurrent output increased to 1.12 μA, and when the water stirring rate was increased to 800 rpm, a 1.98 μA of the current output can be achieved, which is almost 1.7 times higher than the value attained without stirring. The organic pollutant (Rhodamine B; RhB) degradation efficiency using PZT/TiO_2_ particles with UV irradiation and water-stirring was also evaluated (Figure 2C). After 80-min operation with UV irradiation and water-stirring (200 rpm), >75% of the RhB degraded. As the speed of water-stirring increased, the degradation efficiency also increased: 80%, 95%, and 100% of RhB molecules were degraded with the water-stirring speeds of 400, 600, and 800 rpm, respectively. Without UV irradiation, only 25% of RhB was degraded with 800 rpm of water-stirring. In addition, about 32% of RhB was degraded under UV irradiation without any stirring. These controlled experiments further confirmed the synergy mechanisms of the piezo-assisted photocatalysis for AOP water treatment. This work demonstrates a promoting effect where the generated piezoelectric field is used to inhibit photoelectron-hole recombination for high-performance organic pollutants degradation. The PZT/TiO_2_ particles show great potential as solar-induced piezoelectric EHs for harvesting the discrete fluid mechanical energy and solar energy simultaneously, enabling a highly efficient AOP water treatment process.

Instead of using the particle-sized catalysts distributed evenly in water whose application may be limited by the separation process afterward, bulk packaged material with photo-piezo-catalytic effect demonstrates the greater potential for the practical application. As a result, Xu et al. developed a piezoceramic-polymer (i.e., polydimethylsiloxane [PDMS]) porous foam to package the barium strontium titanate (BST) particles to achieve AOP water treatment based on the synergic mechanisms of piezo-assisted photocatalysis (Xu et al., 2020). The developed BST-PDMS porous foam catalyst achieved an enhanced photo-piezo-catalytic coupling effect by combining light irradiation and low-frequency mechanical squeezing (Figure 2D). During the treatment process, the BST-PDMS porous foam was squeezed under a low-frequency (<1 Hz) to mimic the wave or wind press in real-world applications, and a piezoelectric-potential was then generated on the surface of the BST particles. When applied together with the solar irradiation, photo-electron-hole pairs were generated on the BST surface, and charges moved to the surface of the material because the piezoelectric potential inhibited the recombination of electrons and holes. The continuously generated electrons and holes react with O_2_ and water molecules in the solution to produce ·O_2_^-^ and ·OH, respectively, which achieved the AOP water treatment process.

The degradation efficiency of RhB was evaluated using the BST-PDMS porous foam. As shown in Figure 2E, compared to the relatively slow degradation efficiency with either photo irradiation or mechanical squeezing, >90% of RhB degraded when applied with the photo irradiation and mechanical squeezing operations simultaneously within 30 min. The notably enhanced degradation performance confirmed the feasibility of the synergy photo-piezo-catalytic effect for AOP water treatment. Furthermore, the durability and stability of the BST-PDMS porous foam were also evaluated, and 10 cycles for repeatable photo-piezo-catalysis treatment were tested. After 10-cycles treatment, BST-PDMS porous foam degraded by >92.7% RhB after 90 min, which was similar to the degradation efficiency (97.8%) in the first cycle. Thus, the robust packaging process of PDMS porous foam offers improved durability and stability, as well as extends the potential for practical application.

**Figure 3:**
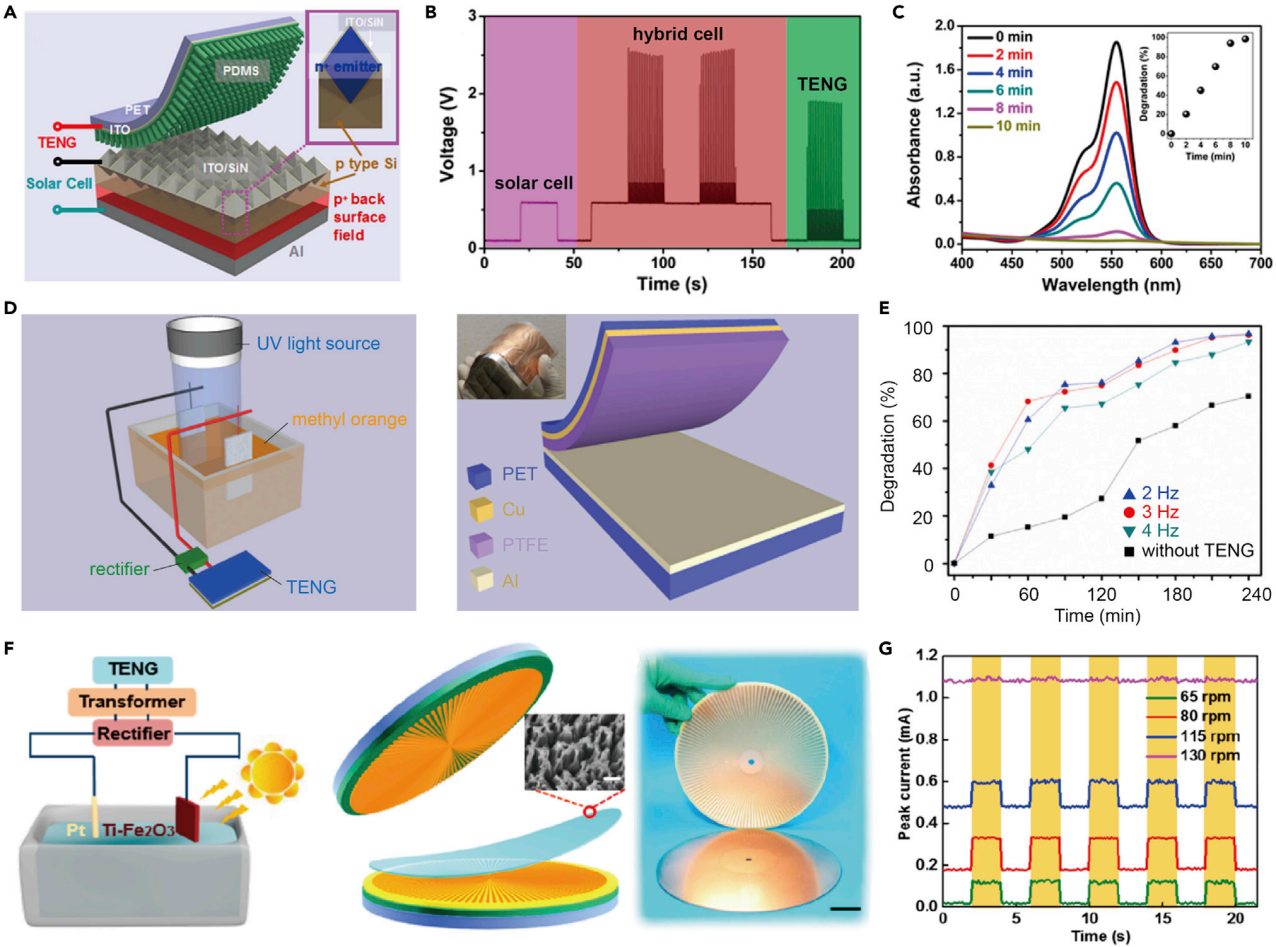
Emerging applications using solar-induced triboelectric EHs for AOP water treatment (A) Schematics showing the structure of the hybrid energy harvesting device integrating triboelectric EHs into solar cells. (B) The output performance (voltage) of the solar-induced triboelectric EH. (C) Absorption spectra of the RhB solution when applying the operation using solar-induced triboelectric EHs. The inset showing the degradation efficiency of RhB with various operation times. Reproduced with permission from (Yang et al., 2013b), Copyright 2013, American Chemical Society. (D) Schematics showing the structure of the UV-induced triboelectric EH for water purification. (E) The degradation efficiency of methyl orange (MO) when applying the operation using UV-induced triboelectric EHs. Reproduced with permission, from (Su et al., 2013), Copyright 2013, IOP Publishing. (F) Schematics showing the potential application using the visible-light-induced triboelectric EH for water purification and structure of the rotational triboelectric energy harvester. (G) Peak current of the EH at the different rotation speeds in the dark or under visible light illumination, showing the potential of using visible-light-induced triboelectric EHs for AOP water treatment. Reproduced with permission from (Wei et al., 2018), Copyright 2018, American Chemical Society.

The piezo-assisted photocatalysis process is also feasible for inactivating microbes in water owing to its enhanced ROS generation mechanisms. Chou et al. developed a molybdenum disulfide (MoS_2_) nanosheets catalyst that can generate ROS to inactivate bacteria through the piezo-assisted photocatalysis process under simultaneous mechanical vibration and light irradiation (Figure 2F) (Chou et al., 2019). The piezoelectric effect of MoS_2_ was investigated using piezo-response force microscopy (PFM). The schematic diagram in Figure 3G showed the formation of the piezoelectric polarization caused by an ambient mechanical force. Once MoS_2_ encountered an eddy force, it experienced unidirectional deformations that induced piezoelectric polarization to inhibit the recombination of the electron-hole pairs generated by photocatalysis. Owing to the internal electric field created by the piezoelectric polarization, the separated electron-hole pairs migrated to opposite sides constantly. The topography and output piezoelectric potential response in the PFM measurement indicated that the active edge sites of MoS_2_ had a comparatively higher output piezoelectric potential than the rest of the material. The positive electric potential on the left (purple color) and the negative electric potential on the right (red color) confirmed the existence of piezoelectric polarization. In addition, the large-scale fabrication of the MoS_2_ nanosheets associated with the potential for practical application was further extended. Using a simple one-step hydrothermal approach, MoS_2_ nanosheets grew uniformly on the surface of carbon fibers (CFs) in a 15 cm × 15 cm area. Moreover, gold nanoparticles (Au NPs) were deposited on MoS_2_ nanosheets to further improve the catalytic efficiency, prolonging the lifetime of electron-hole pairs and enhancing the generation of ROS. When applied with the mechanical vibration and solar light irradiation, the prepared Au-MoS_2_@CFs achieved complete disinfection within 15-min operation (Figure 2H). Attributed to the high ROS generation efficiency caused by the novel piezo-assisted photocatalysis and the feasibility for large-scale application, the Au-MoS_2_@CFs showed great potential to harvest mechanical and solar energy and at the same time offer reliable access to safe drinking water in rural or disaster-stricken areas.

### Solar-induced triboelectric EHs for AOP water treatment

Besides the EHs harvesting mechanical energy based on the piezoelectric effect, triboelectric-based EHs are also confirmed feasible for converting the mechanical energy such as pressing, pushing, as well as wind and flow kinetics energy, effectively (Hinchet et al., 2019; Kim et al., 2020b; Kwak et al., 2019; Yoon et al., 2020). The triboelectric nanogenerator (TENG) is based on the triboelectric effect and electrostatic induction that is capable of harvesting mechanical energy (Ryu et al., 2018; Seol et al., 2018). For this power generation unit, a potential is created in the inner circuit by the triboelectric effect due to charge transfer between two thin organic/inorganic films that exhibit opposite tribo-polarities. In the outer circuit, electrons flow between two electrodes attached on the back sides of the films to balance the potential due to the electrostatic induction. Considering the benefits, broad material availability, lightweight, low cost, high voltage output, and high efficiency for low-frequency stimuli, TENGs are garnering increased interests for application in water treatment (Gao et al., 2017; Jeon et al., 2016; Li et al., 2016; Zhou et al., 2019).

When the solar-induced triboelectric hybrid EH is integrated with solar energy, it shows great potential to drive the AOP water treatment in practical applications. Yang et al. reported on a hybrid EH that integrated a transparent flexible polymer-based TENG and a pyramid patterned silicon (Si) solar cell as one hybrid generator for sustainable and simultaneous energy harvesting (Figure 3A) (Yang et al., 2013b). The transparent polymer-based TENG with a PDMS nanowire array can protect the Si-based solar cell while at the same time harnessing the ambient mechanical energy. The output of this solar-induced triboelectric EH is shown in Figure 3B. The output voltage of the Si-based solar cell device was ~0.6 V. To confirm the feasibility of the solar and triboelectric hybrid nanogenerator with a significantly enhanced hybrid output, the output of the TENG was adjusted in a similar manner to the output from the solar cell by adjusting the TENG structure and applied materials. With the periodic contact and separation operation (i.e., pressing and releasing) between the PDMS nanowire array and the solar cell, the TENG can generate an output voltage of ~2 V after rectification. Furthermore, when applying the solar irradiation and pressing operation simultaneously, the solar-induced triboelectric EH can simultaneously harvest the solar and mechanical energies, respectively, with a synergistic output voltage of ~2.6 V. The feasibility of the developed hybrid EH for driving an electrochemical cell to degrade the pollutants in the water was also investigated. As shown in Figure 3C, the solar-induced triboelectric EH converted the ambient environment energy effectively and achieved a pollutant degradation efficiency of >98% for RhB removal after 10-min of operation using the hybrid EH-powered electrochemical cell. In addition, inside the hybrid energy cell, the solar cell and the rectified TENG device were connected in series, which ensured a sustainable voltage/current output when either the solar energy or mechanical energy is available, which further extends the potential for practical application.

Apart from the solar cell and the TENG hybrid EH, another effective way for water purification is to deploy the TENG to drive the photoelectrochemical water purification process. By using light irradiation, minority electron-hole pairs can be generated on the semiconductor surface, and these generated charges can be driven into the solution by the applied external voltage from TENG to produces ROS for either pollutant degradation or microbial disinfection. First reported by Su in 2013, a contact-separation mode of TENG was developed to drive the photoelectrochemical cell (PEC) for organic pollutant degradation as shown in Figure 3D (Su et al., 2013). When driven by the TENG, the methyl orange (MO) can be degraded effectively with UV irradiation: >95% MO degradation after 240-min of operation as shown in Figure 3E. To further improve the treatment efficiency and extend the application potential, a rotational disc-shaped TENG was developed to harness the flow kinetics for higher output, and a titanium modified hematite photoanode was fabricated to achieve visible light excitation as shown in Figure 3F (Wei et al., 2018). With a relatively slow water flow (~100 rpm rotation speed of the TENG) and solar light (UV irradiation), a notable amount of photocurrent can be achieved (0.6 mA) that correspond to considerable charges driven into the solution, which can be further used for pollutant degradation and microbial disinfection.

### Solar-induced pyroelectric EHs for AOP water treatment

**Figure 4:**
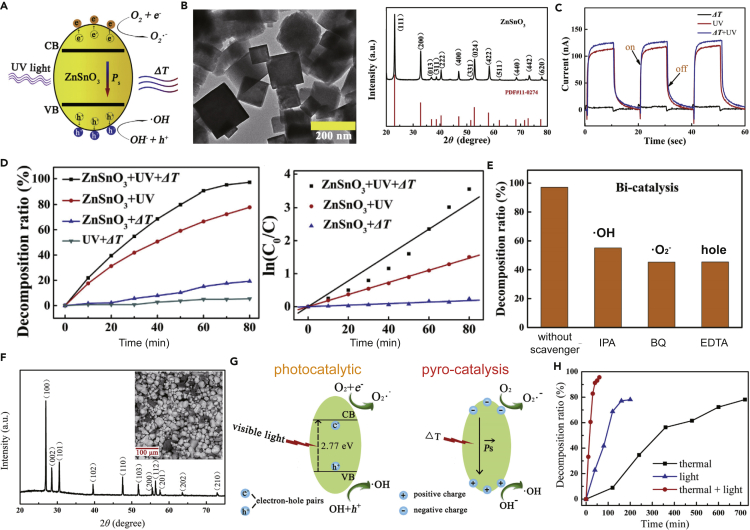
Emerging applications using solar-induced pyroelectric EHs for AOP water treatment (A) Schematics showing ZnSnO_3_ nanoparticles with pyroelectric and photocatalytic features at the same time. (B) Characterizations of ZnSnO_3_ nanoparticles including transmission electron microscope (TEM) image and X-ray diffraction (XRD) pattern. (C) Photocurrent output of ZnSnO_3_ under periodic thermal cycles and solar irradiation. (D) RhB degradation efficiency using ZnSnO_3_ nanoparticles with periodic thermal cycles and solar irradiation. (E) Investigation of the generated ROS during the pyro-assisted photocatalysis degradation. Reproduced with permission, from (Chen et al., 2020), Copyright 2020, Elsevier Ltd. (F) Characterizations of ZnS:Cu nanoparticles including XRD pattern and scanning electron microscope (SEM) image. (G) Mechanism of the photocatalytic and pyro-catalytic dye degradation using ZnS:Cu. (H) Organic pollutant (acid orange 7; AO7) degradation efficiency using ZnS:Cu nanoparticles with solar irradiation and thermal cycles, showing the enhanced performance degradation efficiency based on pyro-assisted photocatalysis mechanisms. Reproduced with permission, from (Luo et al., 2020), Copyright 2020, Elsevier Ltd.

The thermal energy that is abundantly present in the environment and the continuous temperature change can be used as the main driving force of the pyroelectric harvesting systems (Bowen et al., 2014; Wang et al., 2019a; Zhang et al., 2020). Pyroelectric materials can form potential energy on the surface and generate electrical energy based on spontaneous polarization change as a result of the continuous temperature change (Ryu and Kim, 2019; Wang et al., 2017a, 2017b). The generated pyro-potential can inhibit the recombination of the electron-hole pairs for enhanced photocatalysis performance and ROS generation efficiency. Chen et al. developed ZnSnO_3_ nanoparticles that showed pyroelectric and photocatalytic features at the same time (Figure 4A) based on a sol-gel fabrication method as shown in Figure 4B (Chen et al., 2020). As one of the typical ferroelectric lead-free perovskite oxides, ZnSnO_3_ has drawn extensive attention due to its ultra-high remnant polarization. Under the excitation of thermal cycles (20-65°C), the ZnSnO_3_ nanoparticles will polarize, and pyro-potential will be generated on the material surface. At the same time, when applied with UV irradiation, the photo-electron-hole charge pairs will be generated. Owing to the presence of the pyro-potential within the material, the generated electrons and holes will be driven into the solution continuously. The electrons will react with the O_2_ in the water to produce ·O_2_^-^, and holes will oxidize the water molecules to ·OH, and these generated ROSs contribute to AOP water treatment.

To further investigate the synergistic effects of pyro-assisted photocatalysis, the current generated by ZnSnO_3_ was evaluated under various conditions. As shown in Figure 4C, hybrid current with thermal cycles and UV light excitation was superior to that applying only one operation (either thermal cycles or UV irradiation). The enhanced current indicated the synergistic effect between the pyro- and photo-catalysis. The AOP water treatment performance was evaluated by degrading the organic pollution (RhB). With the thermal cycles and UV light excitation, the ZnSnO_3_ achieved high degradation efficiency of >98.1% in RhB removal after 80 min of operation as shown in Figure 4D. This pyro-assisted photocatalytic degradation efficiency was significantly higher than one attained from photo-catalytic (76.8% RhB removal) or pyro-catalytic (20.2% RhB removal). The radical trapping experiment was carried out to explore the synergic mechanisms of pyro-assisted photocatalysis for dye decomposition as indicated in Figure 4E. With the addition of ethylenediaminetetraacetate (EDTA, hole scavenger), benzoquinone (BQ, ·O_2_^-^ scavenger), or isopropanol (IPA, ·OH scavenger), the pyro-assisted photocatalysis performances were slightly suppressed, which suggested the occurrence of holes, ·O_2_^-^, and ·OH during the AOP water treatment process, respectively. As a result, with the benefit of a superior ROS generation efficiency and high energy utilization efficiency, pyro-assisted photocatalysis using ZnSnO_3_ nanoparticles showed great potential in the practical application for AOP water treatment.

Luo et al. (Luo et al., 2020) developed hexagonal wurtzite phase ZnS:Cu nanoparticles, which were also confirmed to be a feasible pyro-assisted photocatalyst for generating ROS for AOP water treatment. ZnS is capable of rapidly generating electron-hole pairs under UV irradiation (sunlight), and the doped transition metal (Cu) can introduce new energy levels into the ZnS bandgap. As a result, the pristine wide bandgap (3.7–3.9 eV) can be reduced to 2.7 eV, making ZnS a visible-light-responsive photocatalyst. Furthermore, the doped Cu molecules can also reduce the symmetry of the host by creating polar domains, causing polarization and pyroelectricity in the host crystals that make ZnS an effective pyro-catalyst (Figure 4F). During the photocatalytic process, the holes are transferred to the new Cu energy levels by radiative transitions, thus preventing the electron-hole recombination and creating superior photocatalytic effects. At the same time, under thermal cycles, these positive and negative charges are released with varied polarization intensity. The generated electrons and holes react with O_2_ and H_2_O to generate the ·O_2_^-^ and ·OH, respectively, and both ·O_2_^-^ and ·OH show a strong ability for oxidizing the dye molecules (Figure 4G). The degradation efficiency of organic pollutants (acid orange 7; AO7) was tested to investigate the pyro-assisted photocatalysis efficiency. After running the operation for 60 min with solar irradiation and thermal cycles simultaneously, >95% AO7 was removed, whereas when only one option was used, only 45% and 10% AO7 was removed under either solar irradiation or thermal cycles. As a result, the ZnS:Cu ceramics exhibit strong pyro-assisted photocatalyst, making it a viable option to run the self-powered AOP water treatment process by utilizing solar and thermal energy effectively.

### Solar-induced thermoelectric EHs for AOP water treatment

Another effective method to harvest thermal energy is by the utilization of EHs based on the thermoelectric effect (He et al., 2018; Shi et al., 2020; Yu et al., 2020; Zhang et al., 2019a). Thermoelectric nanogenerators (TEGs), also called Seebeck generators, are solid-state devices that convert heat flux (temperature differences) directly into electrical energy through a phenomenon called the Seebeck effect (Domnez Noyan et al., 2019; Zhang et al., 2019b). Since its inception, TEG has been used in a wide array of applications ranging from small portable generators to large plant-scale facilities (Yu et al., 2020; Zhao et al., 2019). As a result, when integrated with solar energy, the hybrid solar-induced thermoelectric EHs show great potential to drive the AOP water treatment process and provide reliable access to safe drinking water in rural or disaster-stricken areas.

**Figure 5:**
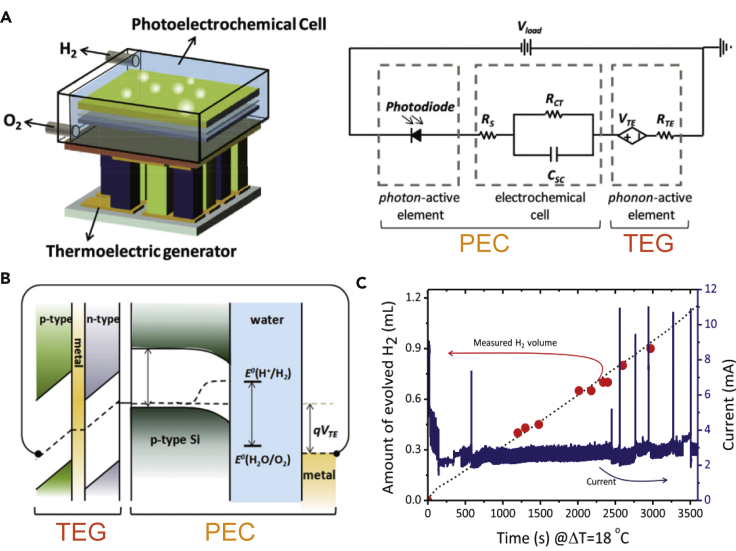
Emerging and potential applications using solar-induced thermoelectric EHs for AOP water treatment (A) Schematics of a hybrid device consisting of a photoelectrochemical cell (PEC) and a thermoelectric nanogenerator (TEG). (B) Illustrations of the generated potential from TEG adjusting the Fermi level of PEC electrode for an enhanced electrons/holes generation efficiency. (C) H_2_ generation efficiency due to the reduction reaction between electrons and water molecules, which can be an essential indicator to confirm the feasibility of ROS generation and AOP water treatment. Reproduced with permission, from (Shin et al., 2015), Copyright 2015, Elsevier Ltd.

Shin et al. developed a catalyst-free PEC cell electrically coupled in series with a TEG, which was capable of utilizing the full solar spectrum by synergistically collecting photon and phonon energies as shown in Figure 5A (Shin et al., 2015). P-type Si (working) and a Pt electrode (counter) were adopted as photocathode and anode for generating hydrogen and oxygen, respectively. Since this work focused only on the enhanced reaction performances of Si photocathode driven by thermo-voltages, only the hydrogen volume generated by the photocathode was measured. To collect hydrogen gas only, a specific system with fritted glass tube isolating a Pt counter electrode was set up. During solar light irradiation, the working cathode of PEC would be triggered, and the solar thermal energy would drive the TEG for a thermo-voltage. When the TEG was connected to the working cathode and the counter electrode (Pt) of the PEC, the Fermi level shifted toward a more positive potential (downward) as shown in Figure 5B. The solar-induced thermoelectric hybrid EH drove the electrons and holes into the water continuously due to the Fermi level lower than the oxidation/reduction potential of water. The generated electrons split the water molecules to produce H_2_, and the H_2_ generation efficiency was tested to evaluate the catalysis performance, which can be an essential indicator to confirm the feasibility for ROS generation and AOP water treatment as shown in Figure 5C. After 50 min of solar irradiation, 0.9 mL of H_2_ was generated. Although the study mainly focused on deploying a hybrid EH to generate electrons and holes for water splitting (i.e., producing H_2_ and O_2_), the thermoelectric-assisted photocatalysis shows great potential for application in the AOP water treatment if applied with proper catalysis and reactor design.

**Figure 6:**
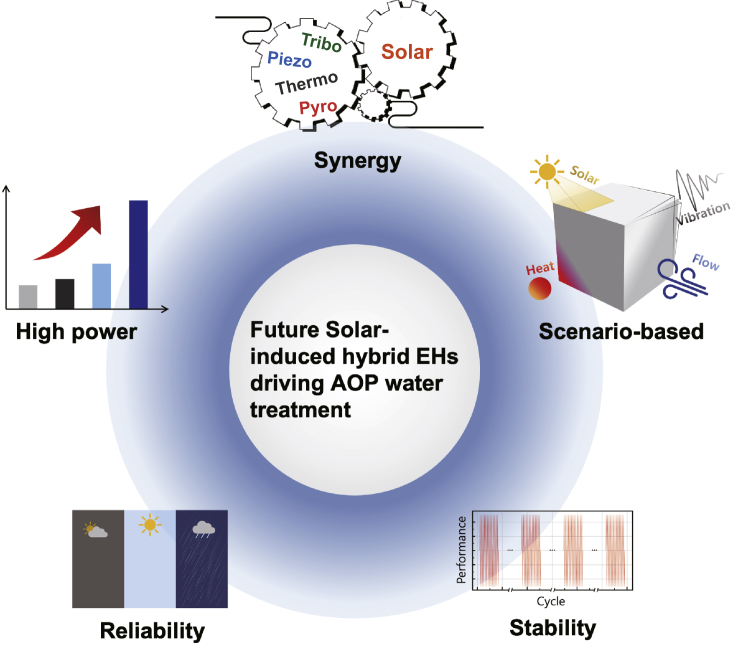
Outlook of research using solar-induced hybrid EHs to drive AOP water treatment technologies

## Prospects and challenges

Energy harvesting systems are designed to convert multiple ambient environment energies, including mechanical and thermal energy. Assisted by sufficient solar energy, the solar-induced hybrid EHs can achieve sustainable, continuous, reliable, and robust energy harvesting, which shows great potential to drive the AOP water treatment. Without any external power input, the solar-induced hybrid EH-driven AOP water treatment methods are capable of purifying drinking water in rural areas where access to clean water and power supplies are limited, and/or during urgent natural disasters and pandemics (e.g., earthquake and COVID-19).

Further advancement of solar-induced hybrid EH-driven AOP water treatment methods will require the development of innovative mechanisms and techniques to achieve synergic processes for more effective water purification processes (Figure 6). Given that energy harvesting processes generate charges or achieve polarization on the material surface, multiple energy harvesting processes can be applied simultaneously for enhanced ROS generation performance. Previous studies have achieved hybrid piezo- and pyro-assisted photocatalysis processes, which show enhanced water purification efficiency with the synergic mechanisms. As a result, other hybrid energy harvesting materials and devices based on the triboelectric effects can be integrated into the photocatalysis process for high-performance AOP water treatment. In addition, to guarantee optimal AOP water treatment performance in practical application with various environmental conditions, the hybrid solar-induced EHs should be designed based on the application scenarios. The rational design of the EHs based on the available energy source (e.g., mechanical, and thermal energy) and harvester layout can further extend the potential for driving the AOP water treatment methods in practical application. In addition, there is still limited research on the solar-induced pyroelectric and thermoelectric EHs for AOP environmental treatment, more attention should be focused on developing novel mechanisms that will drive environmental purification processes and/or devices. Considering the abundance of thermal energy in the ambient environment, solar-induced pyroelectric and thermoelectric EHs have great potential to drive the AOP water treatment processes.

The reliability of the EHs during the AOP water treatment process should further be considered (Xiong et al., 2018; Xu et al., 2019). In practical application, the environmental conditions are usually unstable and changeable with significant humidity and temperature fluctuation, which may affect the efficiency of the energy harvesting process. Most of the time, humidity and other environmental conditions can negatively impact on EHs, especially the triboelectric-based EHs (Wen et al., 2019; Xia et al., 2020a). A humidity-resisting TENG has been developed by applying a layer of electrospun nanofibrous membranes, which eliminates the adverse effects of water vapor on the electrical output (Shen et al., 2017). In addition, the stability, and the output performance of the solar-induced hybrid EHs are equally essential in drive the AOP water treatment process, which can be further improved through the development of material technologies and device design. Provided such advancements, the demonstrated properties of solar-induced hybrid EHs presents significant opportunities for driving the AOP water treatment devices in rural communities and/or during urgent natural disasters and pandemics, which can provide reliable access to safe drinking water and ultimately improve quality of life.

## References

[bib1] Abdelraheem W.H.M., Nadagouda M.N., Dionysiou D.D. (2020). Solar light-assisted remediation of domestic wastewater by NB-TiO_2_ nanoparticles for potable reuse. Appl. Catal. B Environ..

[bib2] Ahmed W., Angel N., Edson J., Bibby K., Bivins A., O’Brien J.W., Choi P.M., Kitajima M., Simpson S.L., Li J. (2020). First confirmed detection of SARS-CoV-2 in untreated wastewater in Australia: a proof of concept for the wastewater surveillance of COVID-19 in the community. Sci. Total Environ..

[bib3] Alvarez P.J.J., Chan C.K., Elimelech M., Halas N.J., Villagrán D. (2018). Emerging opportunities for nanotechnology to enhance water security. Nat. Nanotechnol..

[bib4] Bach C., Dauchy X., Chagnon M.C., Etienne S. (2012). Chemical compounds and toxicological assessments of drinking water stored in polyethylene terephthalate (PET) bottles: a source of controversy reviewed. Water Res..

[bib5] Boczkaj G., Fernandes A. (2017). Wastewater treatment by means of advanced oxidation processes at basic pH conditions: a review. Chem. Eng. J..

[bib6] Bogler A., Packman A., Furman A., Gross A., Kushmaro A., Ronen A., Dagot C., Hill C., Vaizel-Ohayon D., Morgenroth E. (2020). Rethinking wastewater risks and monitoring in light of the COVID-19 pandemic. Nat. Sustain..

[bib7] Bowen C.R., Taylor J., Leboulbar E., Zabek D., Chauhan A., Vaish R. (2014). Pyroelectric materials and devices for energy harvesting applications. Energy Environ. Sci..

[bib8] Cantwell M.G., Katz D.R., Sullivan J.C., Shapley D., Lipscomb J., Epstein J., Juhl A.R., Knudson C., O’Mullan G.D. (2018). Spatial patterns of pharmaceuticals and wastewater tracers in the Hudson River Estuary. Water Res..

[bib9] Cao X., Jie Y., Wang N., Wang Z.L. (2016). Triboelectric nanogenerators driven self-powered electrochemical processes for energy and environmental science. Adv. Energy Mater..

[bib10] Chaplin B.P. (2019). The prospect of electrochemical technologies advancing worldwide water treatment. Acc. Chem. Res..

[bib11] Chen J., Luo W., Yu S., Yang X., Wu Z., Zhang H., Gao J., Mai Y.W., Li Y., Jia Y. (2020). Synergistic effect of photocatalysis and pyrocatalysis of pyroelectric ZnSnO_3_ nanoparticles for dye degradation. Ceram. Int..

[bib12] Chen S., Wang N., Ma L., Li T., Willander M., Jie Y., Cao X., Wang Z.L. (2016). Triboelectric nanogenerator for sustainable wastewater treatment via a self-powered electrochemical process. Adv. Energy Mater..

[bib13] Chen X., Liu L., Feng Y., Wang L., Bian Z., Li H., Wang Z.L. (2017). Fluid eddy induced piezo-promoted photodegradation of organic dye pollutants in wastewater on ZnO nanorod arrays/3D Ni foam. Mater. Today.

[bib14] Cheng Z., Ling L., Wu Z., Fang J., Westerhoff P., Shang C. (2020). Novel visible light-driven photocatalytic chlorine activation process for carbamazepine degradation in drinking water. Environ. Sci. Technol..

[bib15] Chou T.M., Chan S.W., Lin Yu Jiung, Yang P.K., Liu C.C., Lin Yu Jhen, Wu J.M., Lee J.T., Lin Z.H. (2019). A highly efficient Au-MoS2 nanocatalyst for tunable piezocatalytic and photocatalytic water disinfection. Nano Energy.

[bib16] Chu C., Ryberg E.C., Loeb S.K., Suh M.J., Kim J.H. (2019). Water disinfection in rural areas demands unconventional solar technologies. Acc. Chem. Res..

[bib17] De la Obra Jiménez I., López J.L.C., Ibáñez G.R., García B.E., Pérez J.A.S. (2019). Kinetic assessment of antibiotic resistant bacteria inactivation by solar photo-Fenton in batch and continuous flow mode for wastewater reuse. Water Res..

[bib18] Ding W., Zhou J., Cheng J., Wang Z., Guo H., Wu C., Xu S., Wu Z., Xie X., Wang Z.L. (2019). TriboPump: a low-cost, hand-powered water disinfection system. Adv. Energy Mater..

[bib19] Domnez Noyan I., Gadea G., Salleras M., Pacios M., Calaza C., Stranz A., Dolcet M., Morata A., Tarancon A., Fonseca L. (2019). SiGe nanowire arrays based thermoelectric microgenerator. Nano Energy.

[bib20] Feng Y., Li Hao, Ling L., Yan S., Pan D., Ge H., Li Hexing, Bian Z. (2018). Enhanced photocatalytic degradation performance by fluid-induced piezoelectric field. Environ. Sci. Technol..

[bib21] Gao S., Su J., Wei X., Wang M., Tian M., Jiang T., Wang Z.L. (2017). Self-powered electrochemical oxidation of 4-aminoazobenzene driven by a triboelectric nanogenerator. ACS Nano.

[bib22] Guerin T.F. (2017). Evaluating expected and comparing with observed risks on a large-scale solar photovoltaic construction project: a case for reducing the regulatory burden. Renew. Sustain. Energy Rev..

[bib23] Han S.A., Kim T.H., Kim S.K., Lee K.H., Park H.J., Lee J.H., Kim S.W. (2018). Point-defect-passivated MoS_2_ nanosheet-based high performance piezoelectric nanogenerator. Adv. Mater..

[bib24] He M., Lin Y.J., Chiu C.M., Yang W., Zhang B., Yun D., Xie Y., Lin Z.H. (2018). A flexible photo-thermoelectric nanogenerator based on MoS_2_/PU photothermal layer for infrared light harvesting. Nano Energy.

[bib25] Hinchet R., Yoon H.J., Ryu H., Kim M.K., Choi E.K., Kim D.S., Kim S.W. (2019). Transcutaneous ultrasound energy harvesting using capacitive triboelectric technology. Science.

[bib26] Hodges B.C., Cates E.L., Kim J.H. (2018). Challenges and prospects of advanced oxidation water treatment processes using catalytic nanomaterials. Nat. Nanotechnol..

[bib27] Huo Z.Y., Du Y., Chen Z., Wu Y.H., Hu H.Y. (2020). Evaluation and prospects of nanomaterial-enabled innovative processes and devices for water disinfection: a state-of-the-art review. Water Res..

[bib28] Huo Z.Y., Xie X., Yu T., Lu Y., Feng C., Hu H.Y. (2016). Nanowire-modified three-dimensional electrode enabling low-voltage electroporation for water disinfection. Environ. Sci. Technol..

[bib29] Jeon S.B., Kim S., Park S.J., Seol M.L., Kim D., Chang Y.K., Choi Y.K. (2016). Self-powered electro-coagulation system driven by a wind energy harvesting triboelectric nanogenerator for decentralized water treatment. Nano Energy.

[bib30] Kim D., Han S.A., Kim J.H., Lee J.H., Kim S.W., Lee S.W. (2020). Biomolecular piezoelectric materials: from amino acids to living tissues. Adv. Mater..

[bib31] Kim J., Cho H., Han M., Jung Y., Kwak S.S., Yoon H.J., Park B., Kim Hyeok, Kim Hyoungjae, Park J., Kim S.W. (2020). Ultrahigh power output from triboelectric nanogenerator based on serrated electrode via spark discharge. Adv. Energy Mater..

[bib32] Kwak S.S., Kim S.M., Ryu H., Kim J., Khan U., Yoon H.J., Jeong Y.H., Kim S.W. (2019). Butylated melamine formaldehyde as a durable and highly positive friction layer for stable, high output triboelectric nanogenerators. Energy Environ. Sci..

[bib33] Lee J.H., Kim J., Kim T.Y., Al Hossain M.S., Kim S.W., Kim J.H. (2016). All-in-one energy harvesting and storage devices. J. Mater. Chem. A.

[bib34] Lee J.H., Lee K.Y., Kumar B., Tien N.T., Lee N.E., Kim S.W. (2013). Highly sensitive stretchable transparent piezoelectric nanogenerators. Energy Environ. Sci..

[bib35] Lee Ju Hyuck, Yoon H.J., Kim T.Y., Gupta M.K., Lee J.H., Seung W., Ryu H., Kim S.W. (2015). Micropatterned P(VDF-TrFE) film-based piezoelectric nanogenerators for highly sensitive self-powered pressure sensors. Adv. Funct. Mater..

[bib36] Li G.Q., Huo Z.Y., Wu Q.Y., Lu Y., Hu H.Y. (2018). Synergistic effect of combined UV-LED and chlorine treatment on Bacillus subtilis spore inactivation. Sci. Total Environ..

[bib37] Li Z., Chen J., Zhou J., Zheng L., Pradel K.C., Fan X., Guo H., Wen Z., Yeh M.H., Yu C., Wang Z.L. (2016). High-efficiency ramie fiber degumming and self-powered degumming wastewater treatment using triboelectric nanogenerator. Nano Energy.

[bib38] Lin H., Niu J., Liang S., Wang C., Wang Y., Jin F., Luo Q., Huang Q. (2018). Development of macroporous Magnéli phase Ti_4_O_7_ ceramic materials: as an efficient anode for mineralization of poly- and perfluoroalkyl substances. Chem. Eng. J..

[bib39] Liu C., Kong D., Hsu P.C., Yuan H., Lee H.W., Liu Y., Wang H., Wang S., Yan K., Lin D. (2016). Rapid water disinfection using vertically aligned MoS_2_ nanofilms and visible light. Nat. Nanotechnol..

[bib40] Liu C., Min Y., Zhang A.Y., Si Y., Chen J.J., Yu H.Q. (2019). Electrochemical treatment of phenol-containing wastewater by facet-tailored TiO_2_: efficiency, characteristics and mechanisms. Water Res..

[bib41] Liu H., Ni X.Y., Huo Z.Y., Peng L., Li G.Q., Wang C., Wu Y.H., Hu H.Y. (2019). Carbon fiber-based flow-through electrode system (FES) for water disinfection via direct oxidation mechanism with a sequential reduction-oxidation process. Environ. Sci. Technol..

[bib42] Liu S., Ke J., Sun H., Liu J., Tade M.O., Wang S. (2017). Size dependence of uniformed carbon spheres in promoting graphitic carbon nitride toward enhanced photocatalysis. Appl. Catal. B Environ..

[bib43] Loeb S.K., Alvarez P.J.J., Brame J.A., Cates E.L., Choi W., Crittenden J., Dionysiou D.D., Li Q., Li-Puma G., Quan X. (2019). The technology horizon for photocatalytic water treatment: sunrise or sunset?. Environ. Sci. Technol..

[bib44] Luo W., Ying J., Yu S., Yang X., Jia Y., Chen M., Zhang H., Gao J., Li Y., Mai Y.W., Wu Z. (2020). ZnS:Cu powders with strong visible-light photocatalysis and pyro-catalysis for room-temperature dye decomposition. Ceram. Int..

[bib45] Miklos D.B., Remy C., Jekel M., Linden K.G., Drewes J.E., Hübner U. (2018). Evaluation of advanced oxidation processes for water and wastewater treatment - a critical review. Water Res..

[bib46] Moreira F.C., Boaventura R.A.R., Brillas E., Vilar V.J.P. (2017). Electrochemical advanced oxidation processes: a review on their application to synthetic and real wastewaters. Appl. Catal. B Environ..

[bib47] Nie C., Shao N., Wang B., Yuan D., Sui X., Wu H. (2016). Fully solar-driven thermo- and electrochemistry for advanced oxidation processes (STEP-AOPs) of 2-nitrophenol wastewater. Chemosphere.

[bib48] Pan L., Sun S., Chen Y., Wang P., Wang J., Zhang X., Zou J.J., Wang Z.L. (2020). Advances in piezo-phototronic effect enhanced photocatalysis and photoelectrocatalysis. Adv. Energy Mater..

[bib49] Park K. Il, Son J.H., Hwang G.T., Jeong C.K., Ryu J., Koo M., Choi I., Lee S.H., Byun M., Wang Z.L., Lee K.J. (2014). Highly-efficient, flexible piezoelectric PZT thin film nanogenerator on plastic substrates. Adv. Mater..

[bib50] Park S.J., Seol M.L., Kim D., Jeon S.B., Choi Y.K. (2016). Triboelectric nanogenerator with nanostructured metal surface using water-assisted oxidation. Nano Energy.

[bib51] Pooi C.K., Ng H.Y. (2018). Review of low-cost point-of-use water treatment systems for developing communities. Npj Clean. Water.

[bib52] Pu X., Li L., Song H., Du C., Zhao Z., Jiang C., Cao G., Hu W., Wang Z.L. (2015). A self-charging power unit by integration of a textile triboelectric nanogenerator and a flexible lithium-ion battery for wearable electronics. Adv. Mater..

[bib53] Qi C., Yu G., Huang J., Wang B., Wang Y., Deng S. (2018). Activation of persulfate by modified drinking water treatment residuals for sulfamethoxazole degradation. Chem. Eng. J..

[bib54] Qian W., Yang W., Zhang Y., Bowen C.R., Yang Y. (2020). Piezoelectric materials for controlling electro-chemical processes. Nano Micro Lett..

[bib55] Rice J., Westerho P. (2017). High levels of endocrine pollutants in US streams during low flow due to insufficient wastewater dilution. Nat. Geosci..

[bib56] Rizzo L., Agovino T., Nahim-Granados S., Castro-Alférez M., Fernández-Ibáñez P., Polo-López M.I. (2019). Tertiary treatment of urban wastewater by solar and UV-C driven advanced oxidation with peracetic acid: effect on contaminants of emerging concern and antibiotic resistance. Water Res..

[bib57] Rojas J.P., Conchouso D., Arevalo A., Singh D., Foulds I.G., Hussain M.M. (2017). Paper-based origami flexible and foldable thermoelectric nanogenerator. Nano Energy.

[bib58] Ryu H., Kim S.W. (2019). Emerging pyroelectric nanogenerators to convert thermal energy into electrical energy. Small.

[bib59] Ryu H., Lee J.H., Khan U., Kwak S.S., Hinchet R., Kim S.W. (2018). Sustainable direct current powering a triboelectric nanogenerator: via a novel asymmetrical design. Energy Environ. Sci..

[bib60] Ryu H., Yoon H.J., Kim S.W. (2019). Hybrid energy harvesters: toward sustainable energy harvesting. Adv. Mater..

[bib61] Seol M., Kim S., Cho Y., Byun K.E., Kim H., Kim J., Kim S.K., Kim S.W., Shin H.J., Park S. (2018). Triboelectric series of 2D layered materials. Adv. Mater..

[bib62] Shen J., Li Z., Yu J., Ding B. (2017). Humidity-resisting triboelectric nanogenerator for high performance biomechanical energy harvesting. Nano Energy.

[bib63] Shi X.L., Zou J., Chen Z.G. (2020). Advanced thermoelectric design: from materials and structures to devices. Chem. Rev..

[bib64] Shin S.M., Jung J.Y., Park M.J., Song J.W., Lee J.H. (2015). Catalyst-free hydrogen evolution of Si photocathode by thermovoltage-driven solar water splitting. J. Power Sourc..

[bib65] Song K., Mohseni M., Taghipour F. (2016). Application of ultraviolet light-emitting diodes (UV-LEDs) for water disinfection: a review. Water Res..

[bib66] Su Y., Yang Y., Zhang H., Xie Y., Wu Z., Jiang Y., Fukata N., Bando Y., Wang Z.L. (2013). Enhanced photodegradation of methyl orange with TiO_2_ nanoparticles using a triboelectric nanogenerator. Nanotechnology.

[bib67] Thuy Phuong P.T., Zhang Y., Gathercole N., Khanbareh H., Hoang Duy N.P., Zhou X., Zhang D., Zhou K., Dunn S., Bowen C. (2020). Demonstration of enhanced piezo-catalysis for hydrogen generation and water treatment at the ferroelectric curie temperature. iScience.

[bib68] Tian J., Feng H., Yan L., Yu M., Ouyang H., Li H., Jiang W., Jin Y., Zhu G., Li Z., Wang Z.L. (2017). A self-powered sterilization system with both instant and sustainable anti-bacterial ability. Nano Energy.

[bib69] Wang Q., Bowen C.R., Lewis R., Chen J., Lei W., Zhang H., Li M.Y., Jiang S. (2019). Hexagonal boron nitride nanosheets doped pyroelectric ceramic composite for high-performance thermal energy harvesting. Nano Energy.

[bib70] Wang R.N., Zhang Y., Cao Z.H., Wang X.Y., Ma B., Wu W. Bin, Hu N., Huo Z.Y., Yuan Q. Bin (2019). Occurrence of super antibiotic resistance genes in the downstream of the Yangtze River in China: prevalence and antibiotic resistance profiles. Sci. Total Environ..

[bib71] Wang X., Dai Y., Liu R., He X., Li S., Wang Z.L. (2017). Light-triggered pyroelectric nanogenerator based on a pn-junction for self-powered near-infrared photosensing. ACS Nano.

[bib72] Wang X.Q., Tan C.F., Chan K.H., Xu K., Hong M., Kim S.W., Ho G.W. (2017). Nanophotonic-engineered photothermal harnessing for waste heat management and pyroelectric generation. ACS Nano.

[bib73] Wang W.L., Hu H.Y., Liu X., Shi H.X., Zhou T.H., Wang C., Huo Z.Y., Wu Q.Y. (2019). Combination of catalytic ozonation by regenerated granular activated carbon (rGAC) and biological activated carbon in the advanced treatment of textile wastewater for reclamation. Chemosphere.

[bib74] Wei A., Xie X., Wen Z., Zheng H., Lan H., Shao H., Sun X., Zhong J., Lee S.T. (2018). Triboelectric nanogenerator driven self-powered photoelectrochemical water splitting based on hematite photoanodes. ACS Nano.

[bib75] Wen R., Guo J., Yu A., Zhai J., Wang Z.lin (2019). Humidity-resistive triboelectric nanogenerator fabricated using metal organic framework composite. Adv. Funct. Mater..

[bib76] Westerhoff P., Boyer T., Linden K. (2019). Emerging water technologies: global pressures force innovation toward drinking water availability and quality. Acc. Chem. Res..

[bib77] Xia K., Fu J., Xu Z. (2020). Multiple-frequency high-output triboelectric nanogenerator based on a water balloon for all-weather water wave energy harvesting. Adv. Energy Mater..

[bib78] Xia K., Wu D., Fu J., Hoque N.A., Ye Y., Xu Z. (2020). A high-output triboelectric nanogenerator based on nickel-copper bimetallic hydroxide nanowrinkles for self-powered wearable electronics. J. Mater. Chem. A.

[bib79] Xia K., Wu D., Fu J., Xu Z. (2020). A pulse controllable voltage source based on triboelectric nanogenerator. Nano Energy.

[bib80] Xia K., Zhu Z., Zhang H., Du C., Xu Z., Wang R. (2018). Painting a high-output triboelectric nanogenerator on paper for harvesting energy from human body motion. Nano Energy.

[bib81] Xing M., Xu W., Dong C., Bai Y., Zeng J., Zhou Y., Zhang J., Yin Y. (2018). Metal sulfides as excellent Co-catalysts for H_2_O_2_ decomposition in advanced oxidation processes. Chem.

[bib82] Xiong J., Cui P., Chen X., Wang J., Parida K., Lin M.F., Lee P.S. (2018). Skin-touch-actuated textile-based triboelectric nanogenerator with black phosphorus for durable biomechanical energy harvesting. Nat. Commun..

[bib83] Xu S., Qian W., Zhang D., Zhao X., Zhang X., Li C., Bowen C.R., Yang Y. (2020). A coupled photo-piezo-catalytic effect in a BST-PDMS porous foam for enhanced dye wastewater degradation. Nano Energy.

[bib84] Xu W., Wong M.C., Hao J. (2019). Strategies and progress on improving robustness and reliability of triboelectric nanogenerators. Nano Energy.

[bib85] Yang Y. (2020). Recent advances in the electrochemical oxidation water treatment: spotlight on byproduct control. Front. Environ. Sci. Eng..

[bib86] Yang Y., Zhang H., Lee S., Kim D., Hwang W., Wang Z.L. (2013). Hybrid energy cell for degradation of methyl orange by self-powered electrocatalytic oxidation. Nano Lett..

[bib87] Yang Y., Zhang H., Liu Y., Lin Z.H., Lee S., Lin Z., Wong C.P., Wang Z.L. (2013). Silicon-based hybrid energy cell for self-powered electrodegradation and personal electronics. ACS Nano.

[bib88] Yoon H.J., Kang M., Seung W., Kwak S.S., Kim J., Kim H.T., Kim S.W. (2020). Microdischarge-based direct current triboelectric nanogenerator via accumulation of triboelectric charge in atmospheric condition. Adv. Energy Mater..

[bib89] Yoon H.J., Kim S.W. (2020). Nanogenerators to power implantable medical systems. Joule.

[bib90] Yu B., Duan J., Cong H., Xie W., Liu R., Zhuang X., Wang H., Qi B., Xu M., Wang Z.L., Zhou J. (2020). Thermosensitive crystallization-boosted liquid thermocells for low-grade heat harvesting. Science.

[bib91] Zhang D., Wang Y., Yang Y. (2019). Design, performance, and application of thermoelectric nanogenerators. Small.

[bib92] Zhang D., Zhang K., Wang Yuanming, Wang Yuanhao, Yang Y. (2019). Thermoelectric effect induced electricity in stretchable graphene-polymer nanocomposites for ultrasensitive self-powered strain sensor system. Nano Energy.

[bib93] Zhang X., Wu M., Dong H., Li H., Pan B. (2017). Simultaneous oxidation and sequestration of as(III) from water by using redox polymer-based Fe(III) oxide nanocomposite. Environ. Sci. Technol..

[bib94] Zhang Y., Xie M., Adamaki V., Khanbareh H., Bowen C.R. (2017). Control of electro-chemical processes using energy harvesting materials and devices. Chem. Soc. Rev..

[bib95] Zhang Y., Phuong P.T.T., Roake E., Khanbareh H., Wang Y., Dunn S., Bowen C. (2020). Thermal energy harvesting using pyroelectric-electrochemical coupling in ferroelectric materials. Joule.

[bib96] Zhang Y., Sivakumar M., Yang S., Enever K., Ramezanianpour M. (2018). Application of solar energy in water treatment processes: a review. Desalination.

[bib97] Zhao L., Yang Z., Cao Q., Yang L., Zhang X., Jia J., Sang Y., Wu H.J., Zhou W., Liu H. (2019). An earth-abundant and multifunctional Ni nanosheets array as electrocatalysts and heat absorption layer integrated thermoelectric device for overall water splitting. Nano Energy.

[bib98] Zhou L., Liu D., Li S., Yin X., Zhang Chunlei, Li X., Zhang Chuguo, Zhang W., Cao X., Wang J., Wang Z.L. (2019). Effective removing of hexavalent chromium from wasted water by triboelectric nanogenerator driven self-powered electrochemical system - why pulsed DC is better than continuous DC?. Nano Energy.

[bib99] Zi Y., Lin L., Wang J., Wang S., Chen J., Fan X., Yang P.K., Yi F., Wang Z.L. (2015). Triboelectric-pyroelectric-piezoelectric hybrid cell for high-efficiency energy-harvesting and self-powered sensing. Adv. Mater..

